# First cell-patterning of primary, patient-derived mesenchymal glioblastoma brain cancer cells on parylene-C/SiO_2_ substrates

**DOI:** 10.1371/journal.pone.0352239

**Published:** 2026-08-27

**Authors:** Nicholas G. Mellor, Sylvia A. Chung, Pierrette Michaux, Josiah Firth, Scott E. Graham, Bryan W. Day, Charles P. Unsworth

**Affiliations:** 1 The Department of Engineering Science & Biomedical Engineering, Centre of Neural Engineering & Cell Technologies (CoNECT), The University of Auckland, Auckland, New Zealand; 2 Biomedical Engineering, Faculty of Engineering & Information Technology (FEIT), University of Technology Sydney (UTS), Sydney, Australia; 3 Australian National Fabrication Facility (NSW Node) and School of Electrical Engineering & Telecommunications University of New South Wales, Sydney, Australia; 4 Department of Molecular Medicine and Pathology & the Centre for Brain Research, The University of Auckland, Auckland, New Zealand; 5 QIMR Berghofer Medical Research Institute, Brisbane, Australia; University of Waterloo, CANADA

## Abstract

The mesenchymal subtype of the WHO grade IV Glioblastoma (GBM) is the most common and lethal form of adult brain cancer, characterised by its rapid growth, invasiveness and colonisation. Current standard of care includes surgical resection, chemotherapy and radiotherapy. Despite this, mortality remains dismal with median patient survival of less than 15 months after treatment. For these reasons, the World Health Organisation (WHO) classifies GBM as an incurable disease. To address this challenge, the GBM scientific community are investigating new approaches to gain insights into GBM function and its communication mechanisms. These efforts aim to establish alternative therapies to slow or halt the infiltration of this aggressive cancer. In this article we demonstrate the variability between two adult patient-derived mesenchymal GBM brain cancer cell-lines and show how they can be patterned onto grid networks of biocompatible parylene-C on SiO_2_ substrates. Thus, providing a first step for GBM Ca^2+^ communication to be repeatedly and reliably studied. We determine the node size, node spacing and track length that provides the best cytoplasmic coverage of parylene-C for GBM brain cancer cells using five patterning indices, namely: the parylene-C adhesion index (PAI), SiO_2_ adhesion index (SAI), node nuclei index (NNI), grid quality index (GQI), and overall patterning index (OPI). In addition, we determine the best serum, cell seeding density and incubation period *in vitro* to encourage GBM cell patterning. We illustrate high fidelity of the patterned GBM brain cancer networks and to demonstrate cell functionality we show that the patterned GBM cells respond to ATP stimulus with Ca^2+^ transients. The significance of this work is that we provide a biomaterial platform that enables the detailed investigation of Ca^2+^ communication within GBM tumour networks. Such a platform may enable the GBM scientific community to test new therapeutics that target the Ca^2+^ signalling which is involved in the rapid infiltrative tumour growth, a property which currently makes treatment ineffective.

## 1. Introduction

### 1.1. Glioblastoma, tumour cells & calcium

WHO grade IV Glioblastoma (GBM) is the most common and lethal form of malignant adult brain cancer, characterised by its unrivalled invasiveness and colonisation [1]. The current standard of care is a combination therapeutic approach namely, maximal surgical resection followed by concurrent radiotherapy with chemotherapy. Despite this intensive form of clinical treatment, median survival is still less than 15 months, with only 5% or less of patients surviving beyond 5 years [2]. Although the origin of the GBM tumour cell is unknown, evidence suggests it could be derived from the genetic mutations of a particular glial cell phenotype, the astrocyte precursor cell [3,4]. Glial cells comprise ~50% of the brain, of which astrocytes are predominant, comprising 20–40% of the glial. There is now evidence that GBM tumour cells recruit and reprogram healthy glial cells to facilitate invasion of the normal brain parenchyma. The hijacking of healthy cells involves diverse modalities of communication such as ion-channels [5], cytokines, cell-cell contact via gap junctions and, more recently identified, the release of extracellular vesicles [6–8].

**Fig 1:**
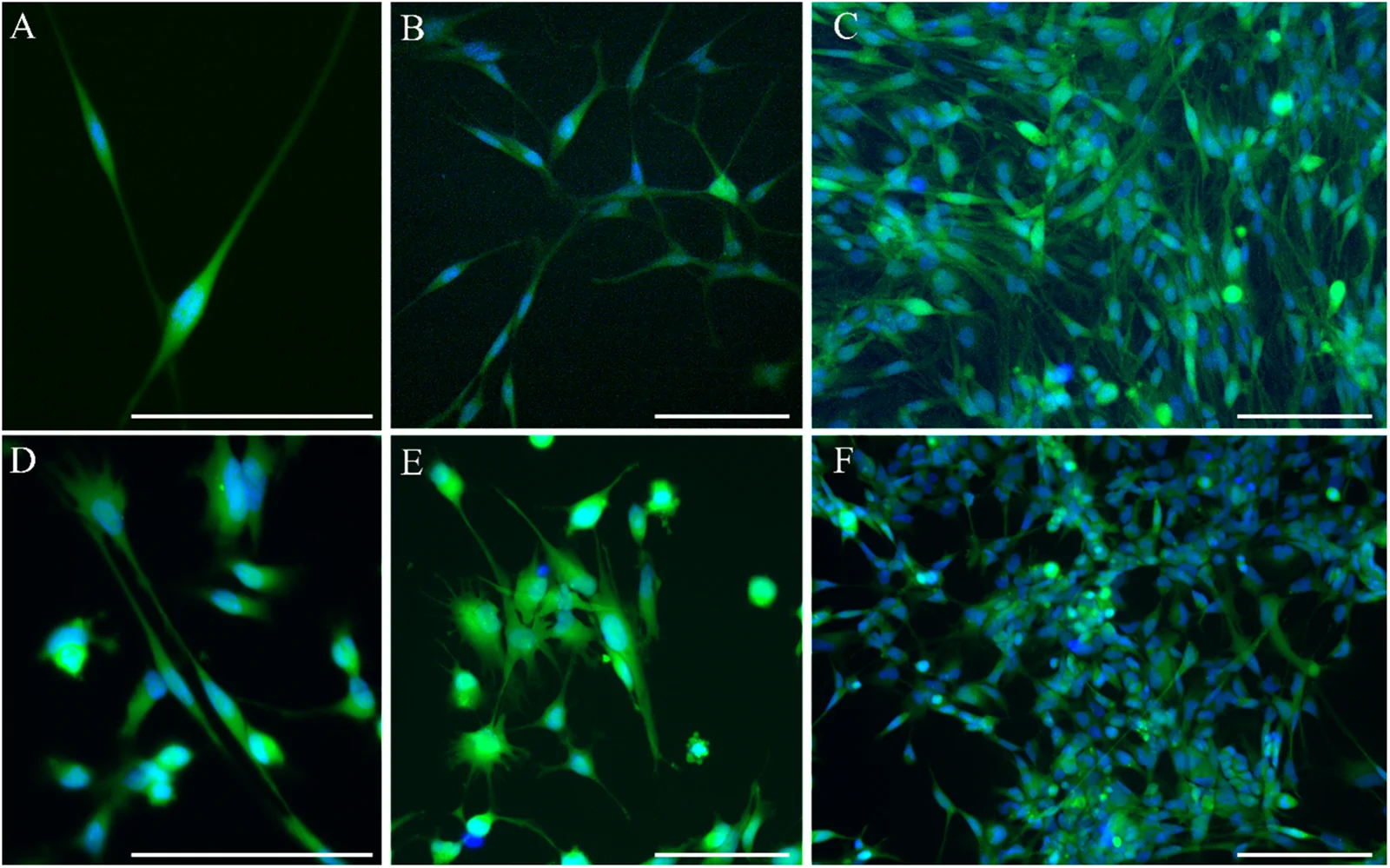
Typical adult patient-derived, mesenchymal GBM brain cancer cells from the FPW1 and RN1 cell-lines grown *in vitro.* (A) A close-up of two FPW1 cells highlighting the general elliptical morphology. (B) and (C) highlight networks of adherent FPW1 cells with multi-layered interconnected network structures. (D) and (E) show small networks of RN1 cells and the multiple morphologies. (F) shows a large network of RN1 cells. All cell bodies (green) are stained with CMFDA and the nuclei (blue) are stained with NucBlue. Scale bars are 100 µm.

Calcium (Ca^2+^) is a fundamental ion messenger involved in a wide range of cellular processes, from cell growth to gene transcription [9]. However, Ca^2+^ channels, which play a critical role in the promotion of in tumourigenesis, can have dramatically altered expressions and activity in GBM [10]. The altered expression of Ca^2+^ channels leads to modified Ca^2+^ signalling that can trigger cell division, angiogenesis, differentiation, and genomic instability in cells [11]. Understanding how Ca^2+^ regulates downstream activity in GBM from single-cells to larger network scales remains unexplored but holds the potential for new therapeutic strategies to cancer treatment. Fig 1 highlights the difficulty in studying Ca^2+^ communication in GBM cells. This is because GBM tumour cells typically grow in an interwoven fashion. Making it challenging to identify the networks they form or how Ca^2+^ communication propagates from the single cells to larger networks, such as those seen in Fig 1C and F.

### 1.2. Cell patterning on parylene-C

Cell patterning [12,13] is a long-established field of neural engineering. It focuses on controlling and directing growth of biological cells. Thus, enabling more effective study of communication within cell networks. Typical forms of patterned networks are regular grid arrays, such that primary modes of communication, like Ca^2+^, can be better observed. Carter first demonstrated cell patterning in 1965 [14] via metal deposition. With the development of photolithography in the 1980s, Kleinfeld pioneered the patterning of neurons [15]. Whitesides demonstrated how soft-lithography could pattern cells, known as ‘micro-contact printing’ [16]. In the early 1990s, Klebe introduced cytoscribing [17], now known as inkjet printing, enabling 3D patterning. Since its inception cell patterning methods have been extended to co-cultures [18,19] and combined with Multi-Electrode Arrays (MEAs) for long-term electrical recording [20].

Parylene-C is a biocompatible material with a variety of desirable properties [21]. Parylene-C’s desirable properties include chemical inertness, electrical insulation, resistance to solvents and acid attack, low water, and gas permeability, and provides a conformal coating through chemical vapour deposition (CVD) [22]. Due to these properties, parylene-C has been widely used in coating printed circuit boards (PCBs) and medical implants [23–25]. In 2009 Delivopoulos [26] demonstrated how fetal rat neurons and glia could be patterned by applying piranha acid to ‘activate’ parylene-C. Activation enabled parylene-C to adsorb and present desirable proteins from equine serum to induce fetal rat neurons to migrate to the parylene-C patterns. Delivopoulos later determined that the ratio of the adhesive glycoprotein fibronectin to albumin proteins played an important role in cell patterning [27]. Delivopoulos also demonstrated how UV photo-oxidation disrupted cell patterning on parylene-C [28]. In 2010, Unsworth patterned primary rat neurons and glial to the single-cell level on ultra-thin parylene-C [29]. In 2011, Unsworth extended this work to the patterning of human brain (NTera2 or hNT) cells on parylene-C using a pre-treatment of fetal bovine serum (FBS) for NTera2 neurons [30] and NTera2 astrocytes [31]. In 2013, Hughes demonstrated how pattern adhesion could be modulated on parylene-C for kidney (HEK293) cells [32]. In 2016, Jordan demonstrated how human NTera2 astrocytic grid networks could be patterned in parylene-C inlayed in SiO_2_ trenches [33]. In 2017, Raos reported the patterning of human NTera2 astrocytes on three other commercially available mainstream parylene derivatives (namely, parylene-HT, parylene-D, and parylene-N) finding that each parylene derivative provides equivalency in cell patterning [34]. In 2018, Raos demonstrated how PEGylating the SiO_2_ substrates could improve the density of human NTera2 astrocytes on parylene-C [35]. In 2019, Li demonstrated how human NTera2 astrocytes could be patterned in large 10x10 single-cell grid networks on parylene-C/SiO_2_ substrates [36]. In 2021, Li demonstrated how different parylene-C micro-shapes could facilitate trackless connections between human NTera2 astrocytes [37]. In June 2021, Delivopoulos demonstrated how neuralised cells derived from mouse embryonic stem cells (mESCs) could be grown successfully on parylene-C/SiO_2_ substrates [38]. Furthermore, parylene-C has been used in other modalities of cell patterning such as: peel-off parylene-C stencils [39–41], and parylene-C neuro-cages for neuron immobilisation [41]. In this study, we will utilise the parylene-C/SiO_2_ platform to pattern patient-derived GBM cells because GBM’s cell of origin is the astrocyte, which has been shown to pattern well on this platform.

Hence, the parylene-C/SiO_2_ platform presented here is aimed at advancing neuro-oncology knowledge by permitting the construction of organised GBM networks. Thus, allowing GBM Ca^2+^ communication to be mapped from the single-cell level through to larger network scales.

## 2. Materials & methods

### 2.1. Design of GBM patterned networks on chip

The parylene-C/SiO_2_ grid networks used in this study were designed using CleWin5™ chip design software. The generic design was a 10 × 10 grid of equally spaced circular nodes connected via thin tracks. The design varied node radius (R_N_) across 5 µm, 8 µm, and 10 µm; and inter-node distance (D_IN_) across 50 µm, 76 µm, and 100 µm. Because GBM processes are very thin, all strip-widths were kept to 2 µm for manufacture which was the smallest feature size to avoid expensive submicron processing costs.

### 2.2. Manufacture of the patterned parylene-C/SiO_2_ chips

**Fig 2:**
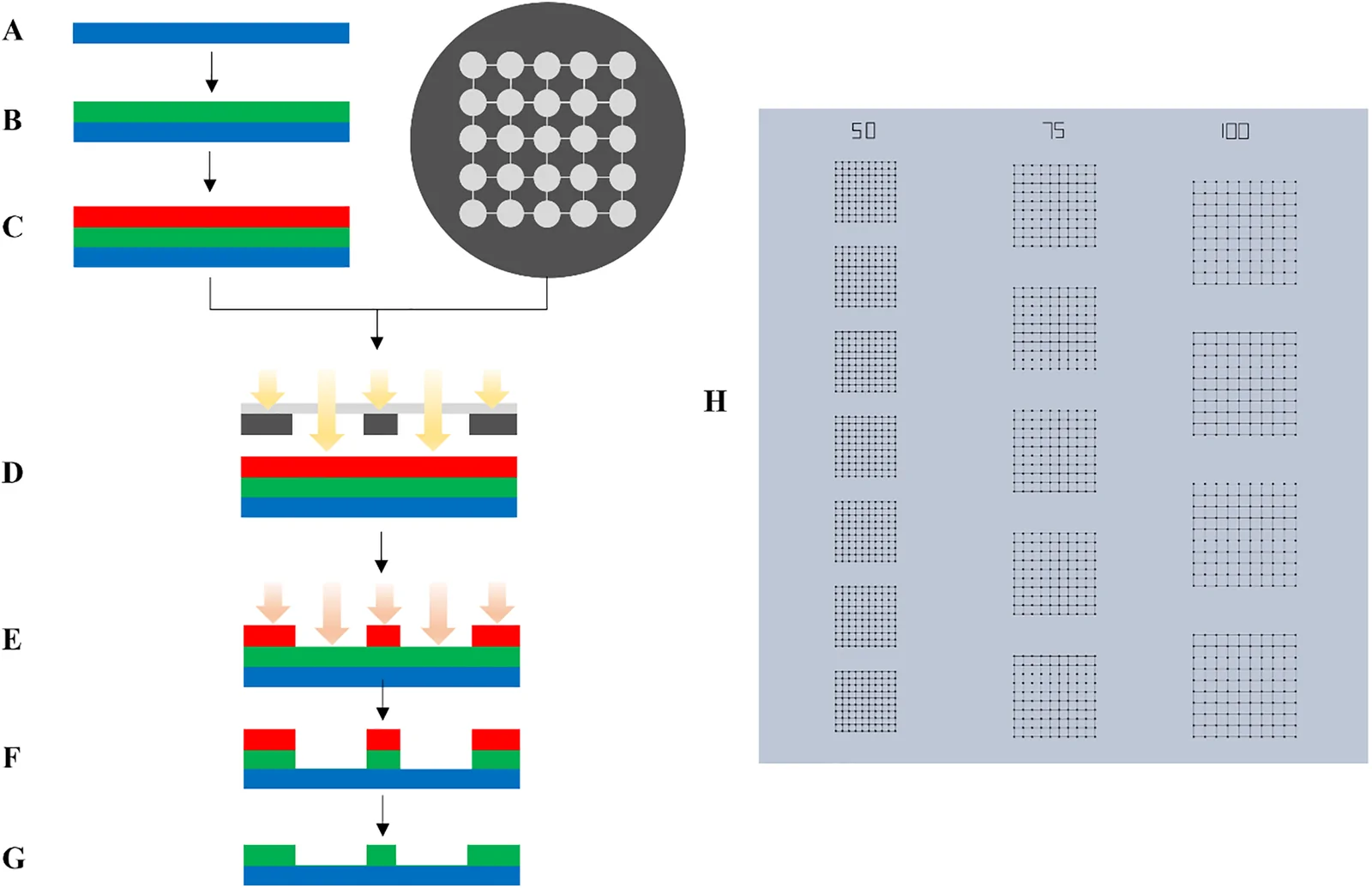
Schematic of the manufacturing process of the chips (not to scale). (A) – (C) the construction of the wafer. The silica wafer (blue) has the layer of parylene-C (green) deposited on top followed by the photo-resistive material (red) to form the layered wafer. (D) The layered wafer and chromium (dark grey) on glass (light grey) mask are then aligned. The layered wafer was exposed to UV light (yellow arrows) through the chromium on glass mask, this irradiates the photo-resistive material in the desired locations. The UV irradiated photo-resistive material was removed thus exposing areas of the underlying parylene-C. (E) Exposed parylene-C was then etched using reactive ion etching (orange arrows). (F) The remaining photo-resistive material is washed off. (G) The final wafer with the pattern is then ready to be diced at the appropriate points. (F) Shows an example chip layout where the R_N_ = 10 µm and the range of D_IN_ values assessed (top of each network pattern columns). Grey represents the SiO_2_ background, black represents the parylene-C network.

The patterned parylene-C/SiO_2_ chips were manufactured using photolithography. This process is shown in Fig 2A-G. A mask of a typical chip with R_N_ = 10 µm is shown in Fig 2H.

1. Wafer preparation: Double-sided polished fused silica wafers were used as substrates (University Wafer). Wafers were soaked for 30 min in a promoter mixture (H2O: IPA: Silane A174/ 100:10:1) for improved parylene adhesion. Wafers were air dried for 30 min, before being soaked in IPA for 5 min and dried with N_2_.
2. Parylene coating: 200 nm of parylene-C was deposited onto the wafer surface at 1.8 nm per g of dimer in the parylene coater system, ParaTech LabTop 3000.
3. Photolithography: A positive tone photo-resist (ECI 3012) was deposited on top of the parylene with a Spin coater POLO 150i at 4000 rpm for 30 s, resulting in a 1.2 um photo-resist layer. A pre-bake at 110°C for 1 min followed. Wafers and photomasks were placed in a Mask Aligner MA6 from Suss, and wafers were exposed at 10 mW cm^-2^ for 1.5 s. Photo-resist was then developed for 2 min in Microchem AZ 826 developer. Resulting in the photo-resist remaining only on the patterning areas.
4. Parylene-C etching: Wafers were placed in a Reactive Ion Etcher to etch unwanted Parylene at 110 nm a minute (at a 20 mT chamber pressure, 15 sccm O2 and 50 W RF power). Parylene-C was removed everywhere but on the patterning areas protected with the photo-resist mask. Wafers were then soaked in Acetone for 2 min to remove residual photo-resist and then rinsed in IPA.
5. Wafer dicing: Chips were produced from the wafer by cutting with a dicing saw (Disco DAD324) at 30,000 rpm and a feed speed of 1 mm s^-1^. The wafers were then packed in a dust free environment for shipping.

### 2.3. Cleaning & preparation of the parylene-C/SiO_2_ chips

Initially, the parylene-C/SiO2 chips were washed in acetone for 30 s to remove residual photo-resist and rinsed in Milli-Q water. Cleaning was then performed by immersing the chips in piranha acid for 15 min. Piranha acid was made using a 5:3 ratio of 30% hydrogen peroxide and 98% sulfuric acid. During piranha acid immersion, air bubbles could form on the chip surface. If this occurred, the chips were gently perturbed to release the bubbles. The chips were rinsed in Milli-Q water and transferred to a sterile cell culture hood. Chips were then placed into 24-well plates and immersed in 2% penicillin-streptomycin-glutamine solution for 40 min. Chips were rinsed with PBS and incubated in three pre-treatments. These pre-treatments were: SFM NSC StemPro media (Gibco, A1050901) (StemPro media), fetal bovine serum (FBS) (Moregate BioTech, FBSF) and PBS (Gibco, 10010023)) all incubated for 3 hours as in [26,33] to identify the treatment that yielded the most effective patterning results. The chips were then ready to be seeded with GBM cells.

### 2.4. GBM cell culture

The primary patient-derived GBM cell-lines, FPW1 and RN1 [42], were used in this study. These were developed and extensively characterised by Stringer et.al [43]. As described in [43], both FPW1 and RN1 were subtyped as mesenchymal, were low-passage, and grown in serum-free conditions [43]. The cell-lines were grown initially on Matrigel (Corning, 354234) coated T75 flasks in StemPro media. The StemPro media was changed every 2–3 days as needed until 90% confluency was obtained, whereupon the cells were harvested for seeding onto the parylene-C/SiO_2_ chips. Cell harvesting was performed by first rinsing the cells with 5 mL of PBS. 1 mL of Accutase (Gibco, A1110501) was then added to the T75 flask for 5 mins to detach the cells from the flask. 1 mL of Defined Trypsin Inhibitor (Gibco, R007100) mixed with 9 mL of PBS was then used to inhibit the Accutase and the cell suspension was collected in a Falcon tube. Next the Falcon tube containing the cell suspension was centrifuged at 200 g for 5 min after which the supernatant was discarded and the cell pellet was resuspended in 1 mL StemPro media. The cells were then ready for seeding onto the parylene-C/SiO_2_ chips. All incubation was performed at 37°C and 5% CO_2_.

### 2.5. Ethics

Institutional ethical approval to use the primary patient-derived cell-lines was granted for this work on 31/3/2021 through the Auckland Health Research Ethics Committee (AHREC), The University of Auckland, New Zealand (Ref. AH22121). The original human patient-derived cell-lines were collected for research purposes on 30/11/2017 by the QIMR Berghofer Medical Research Institute with written informed consent at the Royal Brisbane and Women’s Hospital in accordance with the 2013 version of the 1964 Declaration of Helsinki. The FPW1 and RN1 GBM cell-lines are from male patients and were provided by Bryan Day in June 2021. A full description and characterisation of the GBM cell-lines can be found in [43].

The FPW1 cell-line was accessed for research purposes between 23/08/2022–18/11/2022 and the RN1 cell-line was accessed for research purposes between 06/11/2024–09/12/2024.

### 2.6. Seeding the GBM cells onto the parylene-C/SiO_2_ chips

The resuspended GBM cells were then counted using a hemocytometer (Marinfeld). Cell densities of 100 cells mm^-2^, 200 cells mm^-2^, and 300 cells mm^-2^ were drawn from the cell suspension for seeding onto the 3 different types of treated chip. The chips were aseptically transferred from the 24-well plate to a 48-well plate. Finally, the 1 mL cell suspension was added to the 48-well plate and the chips incubated for 24 h before imaging.

### 2.7. Live cell labelling, imaging & functionality testing

To assess the quality of cell patterning the cells were stained on chip in StemPro media with 1.5 µM of the green live cell membrane dye CMFDA (Invitrogen, C7025) and the blue nuclei dye NucBlue (Invitrogen, R37605) in the 48-well plate. The chips were rinsed twice with imaging media. Imaging media was Fluorobrite™ (Gibco, A1896701) supplemented with 1% GlutaMAX (Gibco, 35050061). Next the chips were transferred to a 35 mm Petri dish which contained 3.5 mL of imaging media. All imaging was performed on an Olympus BX53 upright fluorescence microscope. The microscope had a motorised stage (Marzhauser) and filter set (Olympus). This allowed the GBM patterned networks on the parylene-C/SiO_2_ chips to be imaged. The chips were imaged in DAPI, brightfield, and GFP modalities using a 20x lens by stitching together multiple regularly spaced images via the microscope’s cellSens Dimension (Olympus) software.

To assess the functionality of the patterned GBM cells the cells on chip were stained with 1.5 µM of the Ca^2+^ dye Fluo-4 AM (Invitrogen, F14217), 1.5 µM of the membrane dye CMTPX (Invitrogen, C34552), and NucBlue. They were then rinsed with imaging media twice before being transferred to a polydimethylsiloxane (PDMS) well to which 5 mL imaging media was added. A micro-perfusion system made of a Gilson™ MINIPULS Evolution™ peristaltic pump and microfluidic tubes were then connected to the PDMS well via inlet and outlet holes. Initially, the micro-perfusion system pumped imaging media at a rate of 1 mL min^-2^ for an initial period of 60 s. The pump was then switched to pump imaging media containing 10 µM adenosine triphosphate (ATP) for 30 s before the pump was switched back to imaging media for a further 150 s. Functionality imaging was performed using the GFP modality, and images were acquired at 0.5 Hz using the microscope’s cellSens Dimension software.

### 2.8. Image processing & analysis

All image processing and analysis was performed using MATLAB^®^ (2020b). A nuclei mask was generated from the DAPI image, the membrane mask was generated from the GFP image, the network mask was generated from the brightfield image, and the SiO_2_ mask which was the inverse of the network mask. First a flat-field correction was applied to the stitched images from all three modalities to remove any artifacts from the stitching process and to correct for uneven illumination. Next, the DAPI and GFP images were passed through a 5 × 5 Wiener filter to suppress low-level noise. Then the images were contrast adjusted by histogram equalisation before Otsu’s method [44] was used to threshold these images and generate binary masks. Objects smaller than 100 pixels were removed from the DAPI and GFP masks as cell nuclei and cell bodies are much larger objects. Masks were morphologically closed [44] with a disk structuring element with a radius of 3 pixels to fill any small holes.

**Fig 3:**
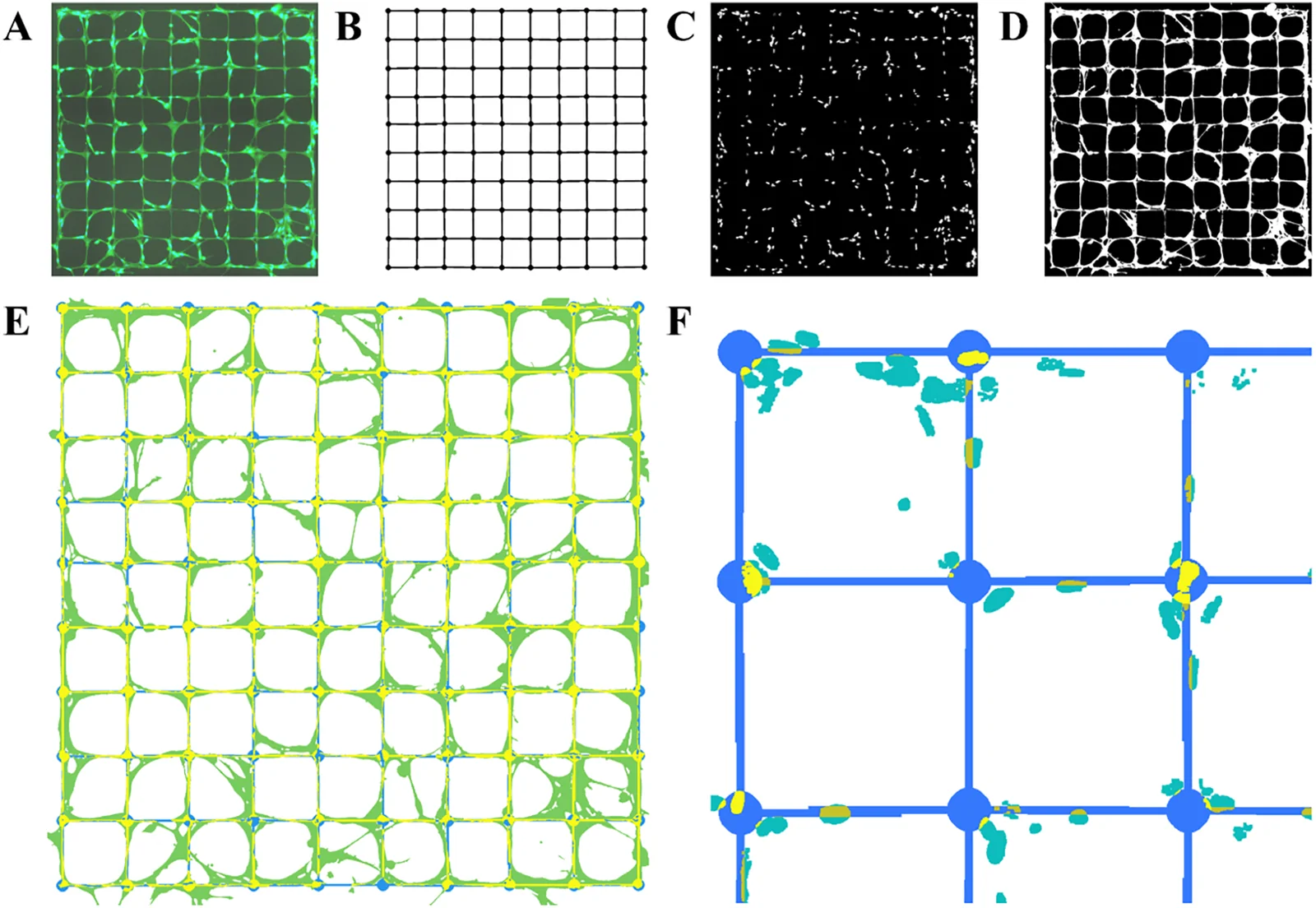
Image processing and analysis workflow. (A) shows a typical fluorescent image of a patterned GBM network. (B) shows the corresponding network mask, (C) the nuclei mask, and (D) the membrane mask. (E) shows graphically PAI (yellow) the amount of cell membrane on the parylene-C, SAI (green) the amount of cell membrane on the SiO_2_, and the network not covered by cell membrane (blue). (F) graphically shows NNI (yellow) the cell nuclei on parylene-C nodes, cell nuclei on SiO_2_ in light blue, cell nuclei on parylene-C strips grey, and the parylene-C in blue.

To determine the quality of the cell patterning four indices were measured: the parylene-C adhesion index (PAI), the SiO_2_ adhesion index (SAI), node nuclei index (NNI), and the grid quality index (GQI) as previously defined [33]. The general workflow of image processing and indices measurement is shown in Fig 3. PAI was a measure of the proportion of cell membrane that was on the parylene-C pattern. PAI was defined as the fraction of the network mask covered by the membrane mask normalised to the total area of the network mask [32]. Thus, PAI ranged from 0 ≤ PAI ≤ 1, where PAI = 0 implied no coverage of the network mask by the membrane mask and PAI = 1 implied complete coverage of the network mask by the membrane mask. SAI measured the proportion of cell membrane that had not patterned and was on the SiO_2_. SAI was defined as the fraction of the SiO_2_ mask covered by the membrane mask normalised to the total area of the SiO_2_ mask. SAI ranged from 0 ≤ SAI ≤ 1, where SAI = 0 implied no coverage of the SiO_2_ mask by the membrane mask, and SAI = 1 implied a complete coverage of the SiO_2_ mask by the membrane mask.

NNI measured the percentage of cell nuclei isolated on parylene-C nodes. NNI was defined as the number of individual objects from the nuclei mask located on the network mask nodes, divided by the total number of objects from the nuclei mask. Therefore, NNI ranged from 0 ≤ NNI ≤ 1. An NNI = 0 implied that there were no cell nuclei on the parylene-C nodes, and an NNI = 1 implied that all cell nuclei were isolated on parylene-C nodes.

Using PAI and SAI, we calculated the grid quality index (GQI) as defined in [37], which measures the quality of the isolation of the cell membrane on the parylene-C and repulsion from the SiO_2_. GQI was calculated as GQI = PAI – SAI and GQI ranged from −1 ≤ GQI ≤ 1. A GQI = 1 implied that the cell membrane lay entirely on the parylene-C and a GQI = 0 implied an equal proportion of cell membrane on the SiO_2_ and the parylene-C network. Similarly, a GQI = −1 implied the cell membrane was entirely localised to the SiO_2_.

Finally, we also defined a new overall patterning index (OPI) as OPI = PAI – SAI + NNI. OPI could range from −1 ≤ OPI ≤ 2. Where an OPI = 2 is defined as optimised when all parylene-C nodes are occupied by cell nuclei only and all parylene-C strips are occupied by cytoplasm only and no cells exist on the SiO_2._ An OPI of −1 means that no cell nuclei exist on the parylene-C nodes and all cytoplasm exists only the SiO_2_. This became a valuable index for this study, as it allows an overall comparison of GBM patterning quality across the range of network dimensions.

## 3. Results

### 3.1. The effect of chip pre-treatments on GBM cell patterning

We assessed the effect of the three pre-treatments that were applied to the parylene-C/SiO_2_ chips on cell patterning. The three pre-treatments were incubation in PBS, StemPro media, and FBS for 3 hours. The cells were then seeded and left to pattern for 1 days *in vitro* (DIV).

**Fig 4:**
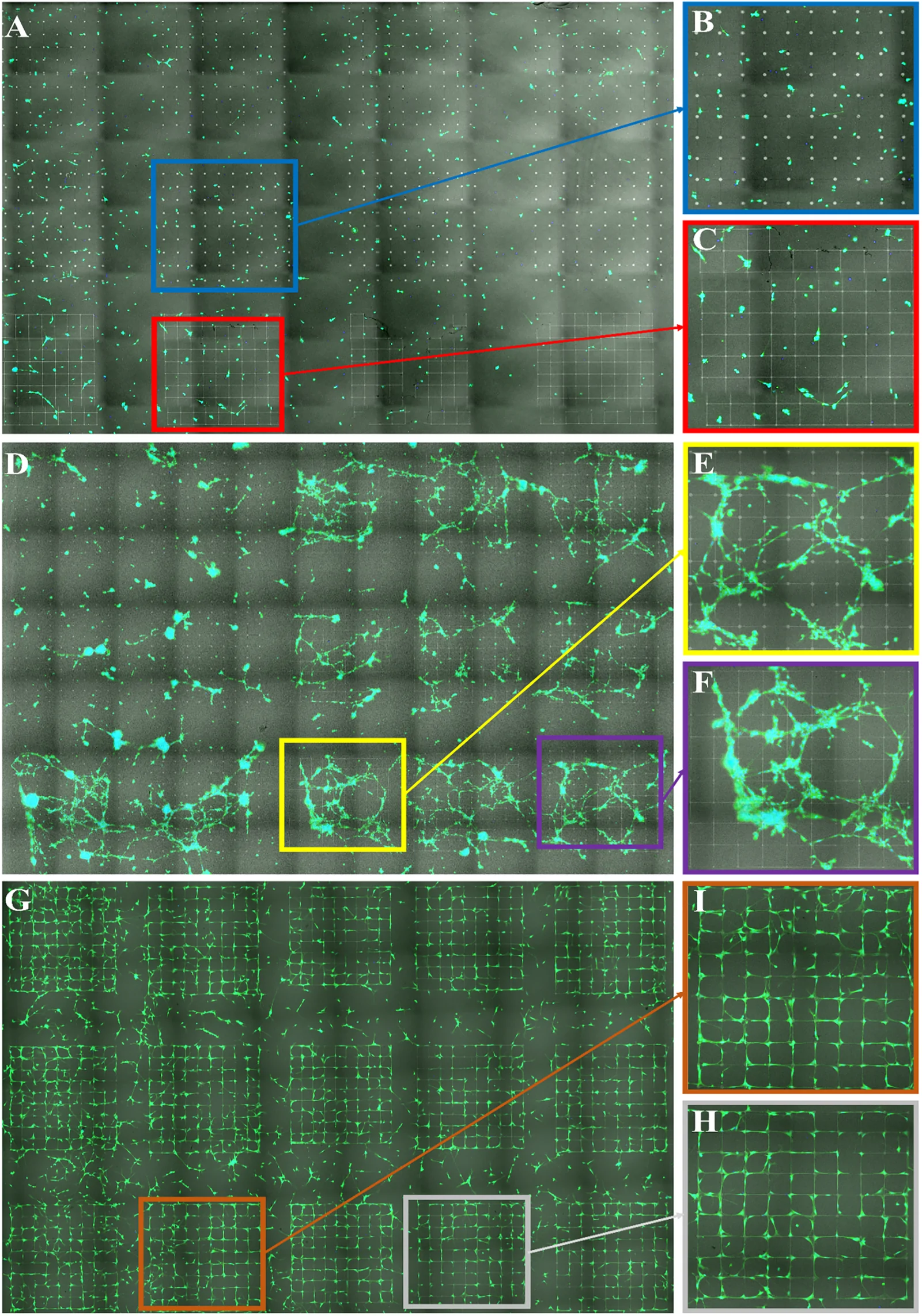
Typical patterning observed for the three chip pre-treatments of incubation in PBS, StemPro media, and FBS for 3 hours. PBS pre-treatment is shown in (A), (B), and (C). StemPro media pre-treatment is shown in (D), (E), and (F). FBS pre-treatment is shown in (G), (H), and (I). Coloured squares correspond to close-ups of typical networks.

The patterning observed on a typical chip incubated in PBS, is shown in Fig 4A, Fig 4B, and Fig 4C. GBM cells on PBS pre-treated chips displayed poor conformity to the underlying pattern. It was observed that the cells did not grow into large clusters and did not prefer to grow within the boundaries of the underlying patterns.

The patterning observed on a typical chip incubated in StemPro media, is shown in Fig 4D, Fig 4E, and Fig 4F. GBM cells seeded onto StemPro pre-treated chips displayed poor conformity to the underlying grid patterns. However, unlike when PBS pre-treatment is used, the GBM cells have grown into large network clusters on the StemPro pre-treated chips. These clusters of GBM cells were observed to preferentially grow within the total boundaries of the underlying patterns as highlighted in Fig 4D.

The patterning observed on a typical chip incubated in FBS, is shown in Fig 4G, Fig 4H, and Fig 4I. Under FBS pre-treatment cells were observed to display conformity to the underlying pattern while also displaying repulsion, although not complete repulsion, from the SiO_2_. Therefore, a pre-treatment of FBS was used to achieve the best patterning results. Untreated parylene-C/SiO2 chips are non-adhesive. Thus parylene-C/SiO_2_ chips must be pre-treated with suitable proteins that encourage cell adhesion. Fig 4 demonstrates that pre-treatment with PBS or StemPro cells does not encourage cell adhesion to the parylene-C as required for organised cell studies.

### 3.2. Determining the optimal network dimensions & seeding density for GBM cell patterning

We assessed patterning across nine different network dimensions, three R_N_ (5 µm, 8 µm, and 10 µm) as it was observed that a typical GBM had a radius of ~10 µm, and three D_IN_ (50 µm, 76 µm, and 100 µm) as it was observed that a GBM process in general extended within these ranges. We also assessed GBM patterning across three seeding densities: 100 cells mm^-2^, 200 cells mm^-2^, and 300 cells mm^-2^.

#### 3.2.1. Visual observations.

**Fig 5:**
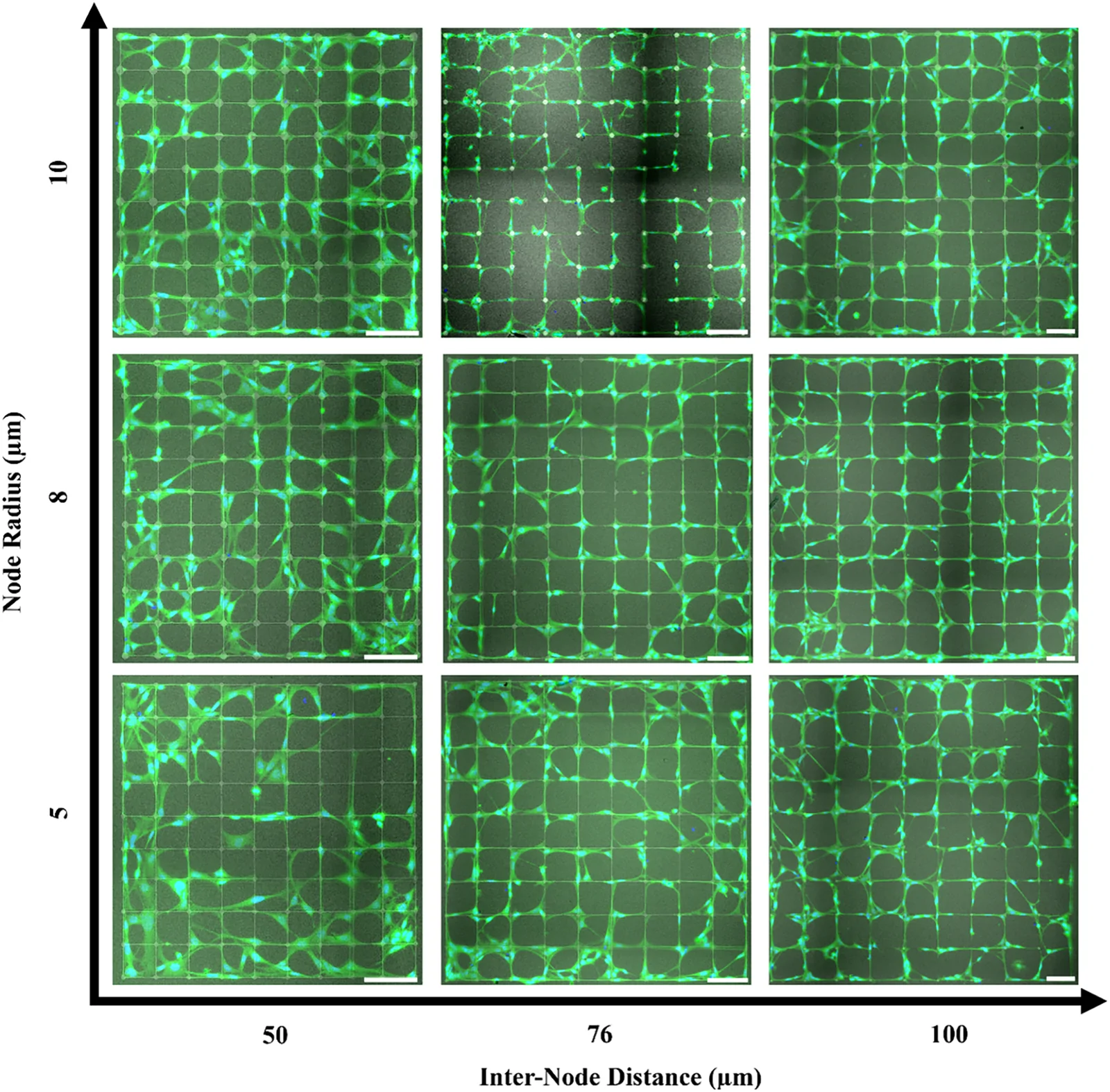
Typical patterned FPW1 cell networks on parylene-C/SiO_2_ substrates seeded at 200 cells mm^-2^. Cell bodies are stained with CMFDA (green) and cell nuclei with NucBlue (blue). All scale bars are 100 µm.

**Fig 6:**
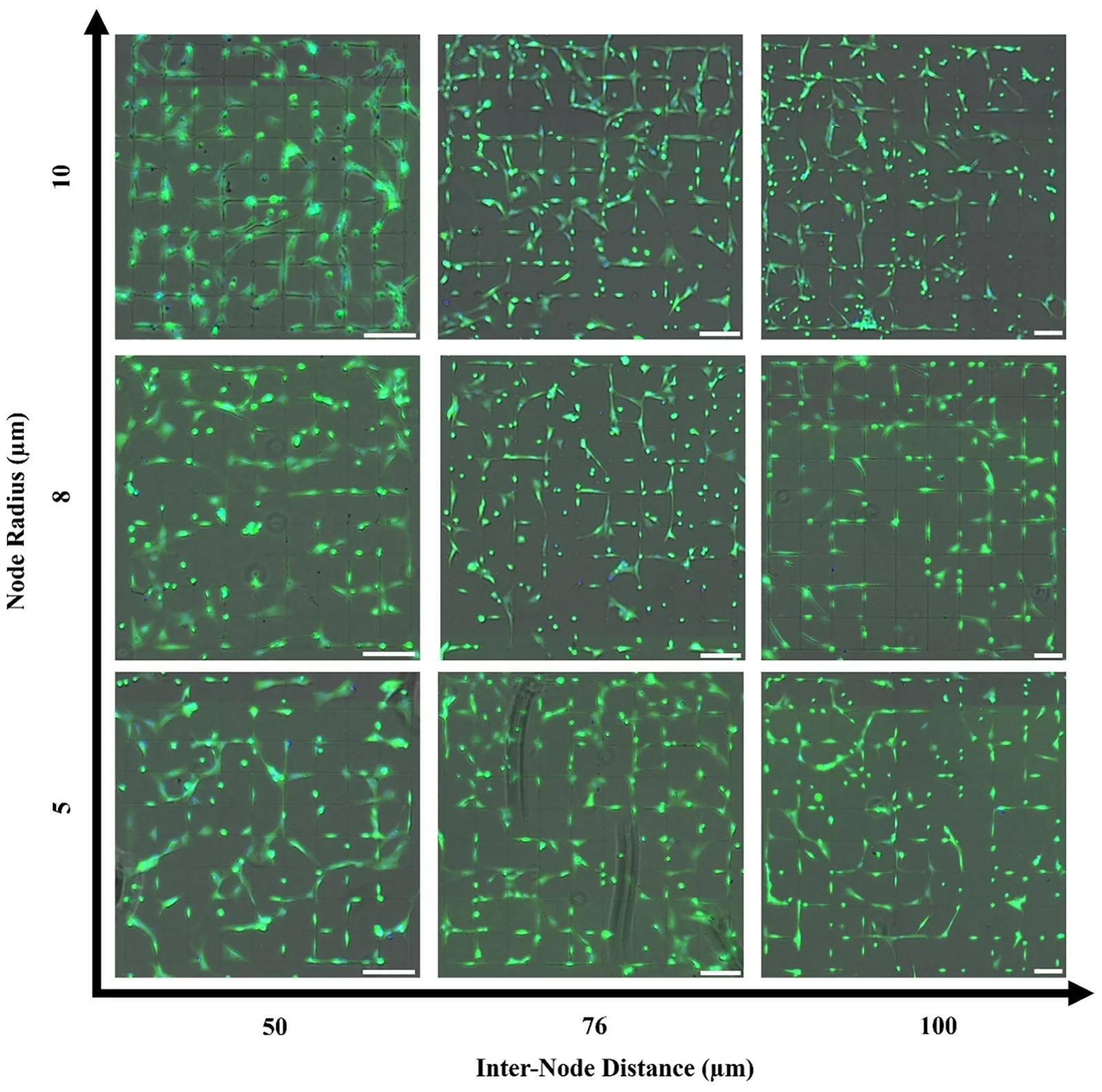
Typical patterned RN1 cell networks on parylene-C/SiO2 substrates seeded at 200 cells mm^-2^. Cell bodies are stained with CMFDA (green) and cell nuclei with NucBlue (blue). All scale bars are 100 µm.

We observed that 200 cells mm^-2^ provided the best patterning for both the FPW1 and RN1 cell-lines which we show in Fig 5 and 6 respectively. Visual observations from Fig 5 show that FPW1 cell patterning was achieved across all network dimensions at a seeding density of 200 cells mm^-2^ although there were patterning differences between the different network dimensions. It could be observed that at D_IN_ = 50 µm, several cells had grown across the SiO_2_ between diagonally adjacent nodes. This suggests that the FPW1 cells tended to grow processes to a length longer than 50 µm, as observed when the FPW1 cells were grown in a Petri dish, Fig 1.

Fig 6, shows that while the RN1 cell-line did conform to the parylene-C it also formed small round cell shapes which did not conform to the parylene-C strips. Visually the coverage of the parylene-C by the RN1 cell-line was lower than the FPW1 cell-line across all the network dimensions.

Typical patterning for the FPW1 and RN1 cell-lines at the seeding densities of 100 cells mm^-2^ to 200 cells mm^-2^ for the different network dimensions are shown in S1–S4 Figs.

#### 3.2.2. Patterning indices.

**Fig 7:**
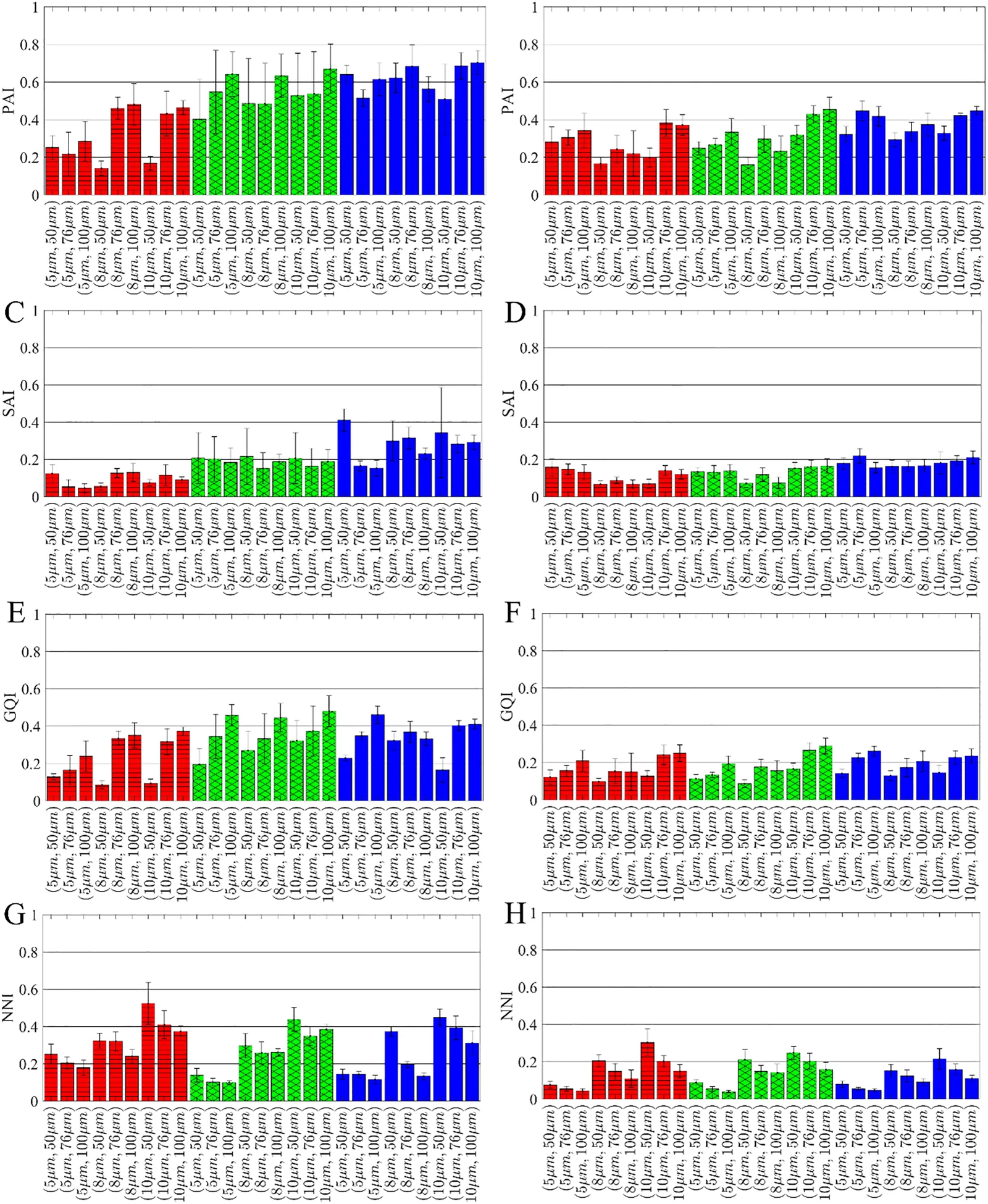
Patterning indices for FPW1 and RN1 cells measured for the different grid dimensions. Red with horizontal hatches, green with cross hatches, and blue with no hatches correspond to a seeding density of 100 cells mm^-2^, 200 cells mm^-2^, and 300 cells mm^-2^, respectively. The indices for the FPW1 cell-line are shown in (A), (C), (E), and (G) and the indices for RN1 cell-line are shown in (B), (D), (F), and (H). PAI (A and B) measures cell membrane coverage of the parylene-C network; SAI (C and D) measures the cell membrane coverage of the SiO_2_; GQI (E and F) measures the cell membrane isolation on parylene-C networks; NNI (G and H) measures the ratio of cell nuclei on parylene-C nodes to the total number of cell nuclei.

**Fig 8:**
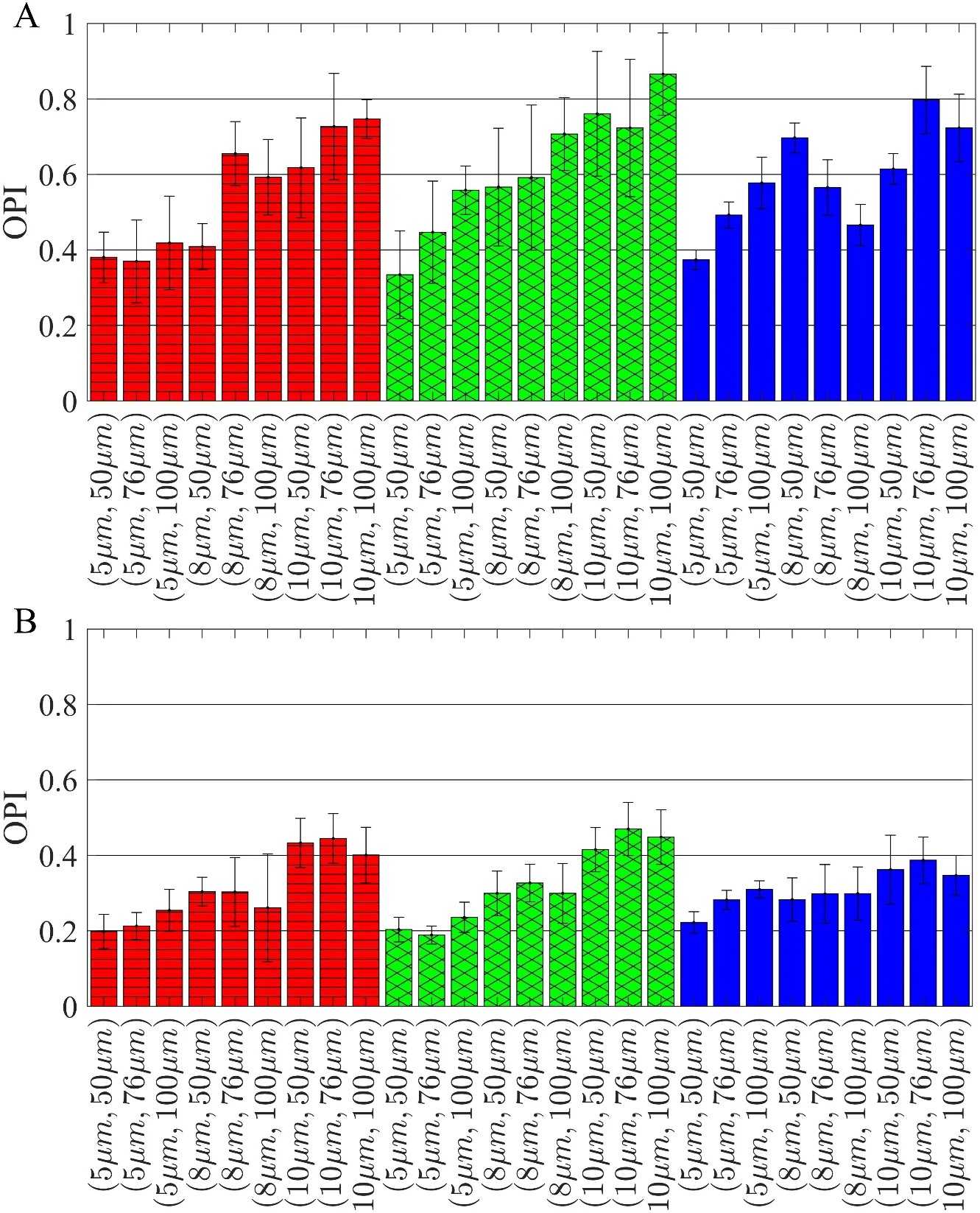
OPI for the FPW1 and RN1 cell-lines shown in (A) and (B) respectively. Red with horizontal hatches, green with cross hatches, and blue with no hatches correspond to a seeding density of 100 cells mm^-2^, 200 cells mm^-2^, and 300 cells mm^-2^, respectively.

The patterning indices PAI, SAI, GQI, and NNI for all network dimensions, and seeding densities are provided in Fig 7 and OPI is provided in Fig 8,with n = 12 for all networks. Red with horizontal hatching corresponds to a seeding density of 100 cells mm^-2^, green with crossed hatching corresponds to a seeding density of 200 cells mm^-2^, and blue corresponds to a seeding density of 300 cells mm^-2^.

PAI is shown in Fig 7A and 7B for the FPW1 and RN1 cell-lines respectively. In general, the FPW1 cell-line had greater PAI values. For the FPW1 cell-line PAI, at a given network dimension, increases with increasing seeding density. However, the effect of increasing FPW1 seeding density on PAI from 100 cells mm^-2^ to 200 cells mm^-2^ is more pronounced than that of increasing from 200 cells mm^-2^ to 300 cells mm^-2^. Whereas the RN1 cell-line PAI was similar at the different seeding densities. At a given seeding density, PAI was most influenced by D_IN_ with most D_IN_ = 100 µm networks having higher PAI values for a given R_N_. FPW1 PAI was the highest, PAI = 0.70 ± 0.06, at a seeding density of 300 cells mm^-2^ for network dimensions D_IN_ = 100 µm and R_N_ = 10 µm. RN1 PAI was the highest, PAI = 0.45 ± 0.06, at a seeding density of 200 cells mm^-2^ for network dimensions D_IN_ = 100 µm and R_N_ = 10 µm.

SAI is shown in Fig 7C and 7D for the FPW1 and RN1 cell-lines respectively. In general SAI at a given network dimension increases with increasing seeding density, this effect is more pronounced with the FPW1 cell-line. The lowest FPW1 SAI at a seeding density of 100 cells mm^-2^ was SAI = 0.05 ± 0.02 and occurred at network dimensions D_IN_ = 100 µm and R_N_ = 5 µm. The lowest RN1 SAI at a seeding density of 100 cells mm^-2^ was SAI = 0.07 ± 0.02 and occurred at network dimensions D_IN_ = 50 µm and R_N_ = 8 µm. In general, the FPW1 cell-line had larger SAI values for a given patterning condition.

GQI is shown in Fig 7E and 7F and reflects PAI minus SAI. GQI for the FPW1 cell-line increases with increasing D_IN_ at most seeding densities and for a given R_N_. The opposite effect is observed with the RN1 cell-line where increasing D_IN_ results in a decrease in GQI for a given R_N_. Overall GQI is higher for the FPW1 cell-line when compared to the RN1 cell-line. The highest FPW1 GQI is 0.48 ± 0.08 and the highest RN1 GQI is 0.29 ± 0.04 both of which occurred at seeding density of 200 cells mm^-2^ and network dimensions of D_IN_ = 100 µm and R_N_ = 10 µm.

NNI is shown in Fig 7G and 7H. Across all seeding densities, FPW1 NNI was most influenced by R_N_. The largest FPW1 NNI of 0.52 ± 0.11 and RN1 NNI of 0.3 ± 0.07, were found to both occur at a seeding density of 100 cells mm^-2^ for network dimensions of D_IN_ = 50 µm and R_N_ = 10 µm.

OPI for FPW1 and RN1 are shown in Fig 8A and 8B respectively. On average the FPW1 cell-line had an OPI that was higher than the RN1 cell-line at a given network dimension. Across both cell-lines OPI generally increased with increasing D_IN_, R_IN_, and seeding density. The highest FPW1 OPI was 0.87 ± 0.11 and occurred at a seeding density of 200 cells mm^-2^ for network dimensions of D_IN_ = 100 µm and R_N_ = 10 µm. The highest RN1 OPI was 0.47 ± 0.07 and occurred at a seeding density of 200 cells mm^-2^ for network dimensions of D_IN_ = 76 µm and R_N_ = 10 µm.

Using OPI we determined the optimal patterning conditions for the FPW1 cell-line to be a cell seeding density of 200 cells mm^-2^ and network dimensions of D_IN_ = 100 µm and R_N_ = 10 µm. For the RN1 cell-line the OPI optimal pattering conditions were found to be a cell seeding density of 200 cells mm^-2^ and network dimensions of D_IN_ = 76 µm and R_N_ = 10 µm.

The most generic patterning parameter, OPI, was used for the statistical tests. Because of the non-Gaussian nature of the OPI data a non-parametric Kruskal Wallis test was used [45]. The Kruskal Wallis test indicated (p << 0.05) that there were statistical differences between the different patterning conditions so Dunn’s test with a Bonferroni correction was used for pairwise comparisons. The multiple comparison for both the FPW1 and RN1 cell-lines are shown in S1 and S2 Table respectively, the comparisons to OPI optimal patterning conditions shown. The patterning conditions which are statistically significantly different (p < 0.05) are highlighted in green. GraphPad Prism 11 was used for the Kruskal Wallis test, Dunn’s test, and Bonferroni correction.

### 3.3. Assessing functionality of GBM patterned networks on chip

**Fig 9:**
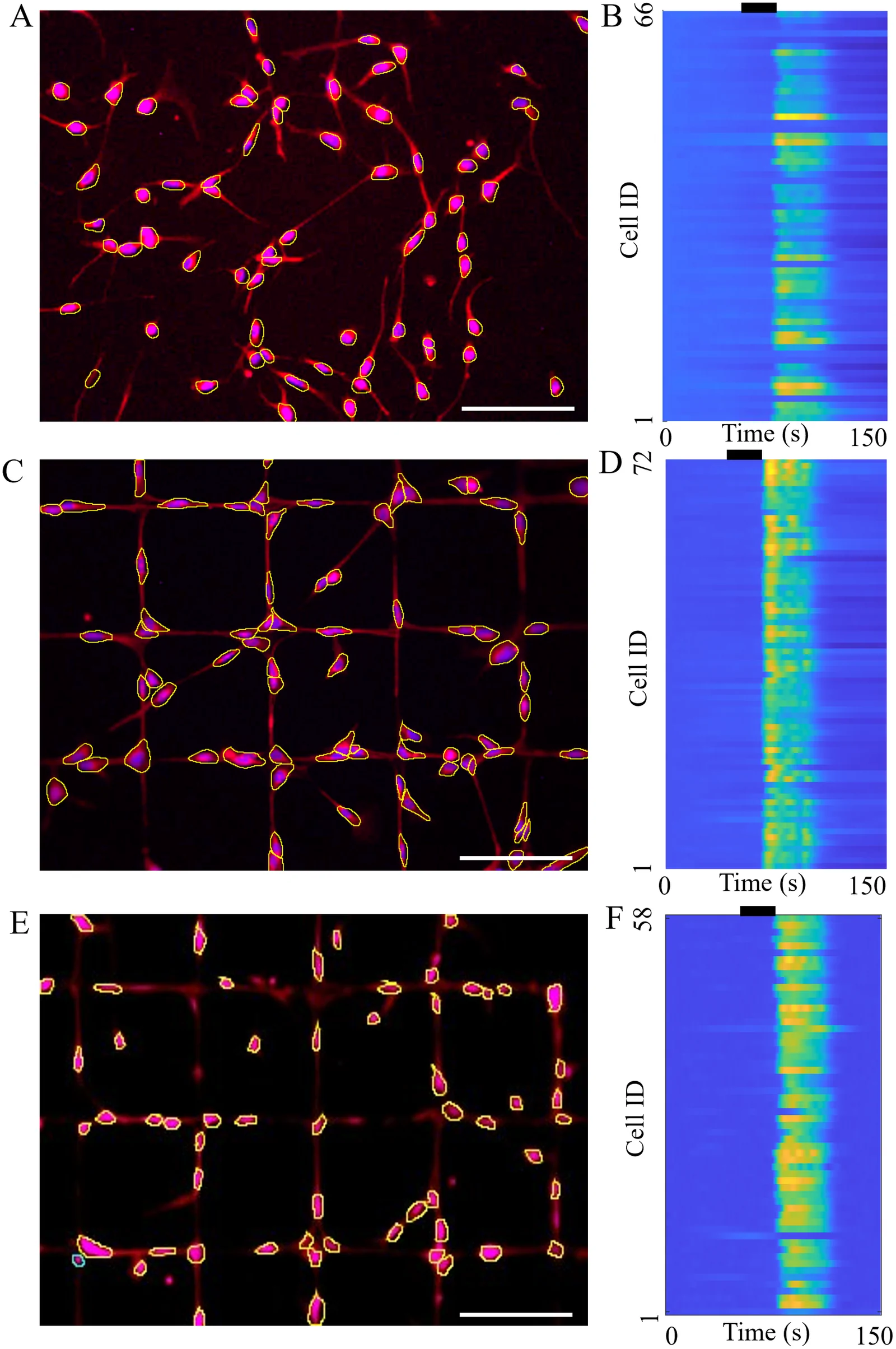
Non-patterned and patterned GBM cell responses to ATP stimulation. (A) shows a fluorescent image of FPW1 cells grown unpatterned in a petri dish. (B) is the corresponding Ca^2+^ heatmap of ATP perfusion over the cells shown in (A). (C) shows FPW1 cells patterned on a parylene-C/SiO_2_ chip with the OPI optimal dimensions. (D) is the corresponding Ca^2+^ heatmap of ATP perfusion over the cells shown in (C). (E) shows RN1 cells patterned on a parylene-C/SiO_2_ chip with the OPI optimal dimensions. (F) is the corresponding Ca^2+^ heatmap of ATP perfusion over the cells shown in (E). Cell bodies are stained with CMTPX (red), the cell nuclei with NucBlue (blue), and the yellow boundaries demarcate the identified individual cells. Scale bars are 100 µm.

We assessed the functionality of the GBM patterned networks by administering ATP which is known to induce Ca^2+^ responses in astrocytes [33,46] and GBM cell-lines [47]. Initially, the functionality of GBM cells under normal conditions in a 35 mm Petri dish was assessed. Fig 9B shows a heatmap of the Ca^2+^ response to ATP stimulus of the FPW1 cells shown in Fig 9A. As imaging media was perfused over the cells for 60 s prior to ATP perfusion we used this period as the negative control period. The 30 s period after the negative control period was the ATP stimulus period. The percentage of cells responding under the different conditions within these periods are shown in Table 1. A cell was deemed to have responded if it had a maximum value over 10% of the average maximum of all the cells in the recording.

**Table 1:** Functionality tests on unpatterned and patterned GBM cells using ATP.

	Unpatterned FPW1	Unpatterned RN1	Patterned FPW1	Patterned RN1
Negative Control Period % Responding	0%	1%	3%	1%
ATP Period % Responding	84%	78%	96%	84%
FWHM ± SD	15.8 ± 3.1 s	15.0 ± 3.6 s	11.8 ± 1.8 s	12.7 ± 5.1 s

Functionality of GBM cells under normal conditions in a 35 mm Petri dish was compared to patterned cells using the full width half maximum (FWHM) which was defined as the time difference between half the maximum of a calcium transient. The FWHM distributions of unpatterned to patterned cells were then compared with a two-sample t-test which was unable to reject the null hypothesis. The average FWHM is shown in Table 1. We did not compare magnitudes of the calcium transients as we used the single wavelength dye Fluo-4. A single wavelength dye makes comparing images on different substrates difficult as there can be differences in background noise and interference. Fig 9C and 9E, show patterned FPW1 and RN1 cells respectively with the corresponding Ca^2+^ response heatmaps shown in Fig 9D and 9F. These cells were patterned on the grid network with dimensions of R_N_ = 10 µm and D_IN_ = 100 µm.

## 4. Discussion

We have demonstrated that two patient-derived mesenchymal GBM cells can be successfully patterned into functional networks on parylene-C/SiO_2_ substrates. This GBM patterning platform can be used to track the flow of Ca^2+^ communication from single cells to large network scales in a repeatable manner.

To demonstrate GBM patterning networks of 10 × 10 grids of equally spaced circular nodes connected via thin tracks were fabricated in parylene-C deposited on SiO_2_ substrates. Two parameters were controlled to determine the grid dimensions that provided the highest fidelity of cell patterning. These were the node radius R_N_ sampled at 5 µm, 8 µm, and 10 µm and inter-node distance D_IN_ 50 µm, 76 µm, and 100 µm as it was observed that GBM cell bodies and processes generally fell within these ranges. We assessed the effect of three pre-treatments applied to the parylene-C/SiO_2_ chips and their ability to influence GBM cell patterning. This was performed by incubating the chips in StemPro media, PBS, and FBS for 3 hours. The cells were then seeded and left to pattern for 1 DIV. We next investigated the effect of seeding density on GBM cell patterning. Seeding densities of 100 cells mm^-2^, 200 cells mm^-2^, and 300 cells mm^-2^ were trialed. Once the best seeding density had been determined, the GBM cells were patterned on the parylene-C/SiO_2_ chips to determine the chip dimensions that resulted in optimal patterning. Patterning quality was assessed by measuring five cell patterning indices PAI, SAI, NNI, GQI, and OPI, with OPI being used to determine the optimal network design. Finally, functionality of GBM cell networks patterned on the OPI optimum network design was tested through measuring Ca^2+^ responses to ATP stimulation.

Each of the findings for the patient-derived, mesenchymal GBM brain cancer cells will now be discussed with a comparison to the existing parylene-C/SiO_2_ literature.

For both GBM cell-lines 3 hour FBS immersion provided the best parylene-C pre-treatment. FBS pre-treatment of parylene-C was consistent with patterning of immortalised human embryonic kidney cells (HEK 293) [34], human NT2 neurons [30,33,36], and human NTera2 astrocytes. On the other hand, primary fetal rat neurons preferred equine serum pre-treatment [27]. In [38], neuralised cells derived from mouse embryonic stem cells (mESCs) were also found to prefer FBS. In addition, FBS was the favored pre-treatment for human NTera2 astrocytes on three commercially available mainstream parylene derivatives (parylene-HT, parylene-D and parylene-N) [34]. Therefore, mesenchymal GBM cells preferred the same FBS pre-treatment as all other human cell types patterned on parylene-C/SiO_2_ substrates. A pre-treatment incubation time of 3 hours for patient-derived, mesenchymal GBM brain cancer cells was also found to be consistent with the patterning of other the cell types [27,32,34,37,40].

A seeding density of 200 cells mm^-2^ was found to maximise the OPI of both patient-derived, mesenchymal GBM cell-lines. NTera2 astrocytes have been patterned with seeding densities of 50 cells mm^-2^ on single square nodes [34], on 10 × 10 networks [36], and 100 cells mm^-2^ on 10 × 10 trench networks [33]. Additionally, NTera2 neurons [30] were found to have maximal parylene-C coverage at a seeding density of 100 cells mm^-2^ when patterned on thin parylene-C strips. Primary rat neurons have been found to produce reliable patterning with seeding densities of 70 cells mm^-2^ [30] and 150 cells mm^-2^ [26] when patterned on thin strips. A seeding density of 150 cells mm^-2^ was used to pattern neuralised mESC onto thin strips [38] and 200 cells mm^-2^ was employed to grow mESC on unpatterned parylene-C [27]. Compared to the patterning of NTera2 astrocytes onto grid networks [37,40], mesenchymal GBM cell-lines required a higher seeding density for patterning. This likely reflects cell-line size differences as NTera2 astrocytes (diameter of ~100 µm) are larger than mesenchymal GBM cells (diameter of ~10 µm). The FPW1 and RN1 GBM cell-lines are a similar size to primary rat neurons [27,30], NTera2 neurons [31], and mESCs [42]. However, these were not patterned on grid networks so it was not possible to make a direct comparison of the seeding density of these cell types. Furthermore, neurons with neurites have a very different cell morphology from astrocytes and the GBM cell-lines, so it would follow that they require different seeding densities.

The OPI optimal network dimensions for patterning FPW1 GBM cells were R_N_ = 10 µm and D_IN_ = 100 µm and for RN1 GBM cells they were R_N_ = 10 µm and D_IN_ = 76 µm. For both cell-lines a strip width of 2 µm was used. Similar OPI optimal network dimensions for the GBM cell-lines likely reflects that the two cell-lines display similar morphologies when grown *in vitro*. As shown in Fig 1 the RN1 cell-line displayed cell morphologies with rounder cell bodies and smaller processes which could explain the lower D_IN_. Maximal parylene-C coverage of NTera2 astrocytes on parylene-C/SiO_2_ networks was found to occur on square nodes with side length of 65 µm, D_IN_ = 90 µm, and a strip width of 5 µm [36]. Mesenchymal GBM cell-lines and NTera2 astrocytes [40] both pattern with D_IN_ = ~ 100 µm. Suggesting that these cells can extend out processes of similar sizes. However, the R_N_ values were found to be significantly different, reflecting that these two cell types have different sized cell bodies. NTera2 astrocyte patterning in trench networks [37] was also found to have the same node size (R_N_ = 10 µm), however, as these nodes were in trenches with a depth of 30 µm a direct comparison is not possible. Additionally, the patient-derived, mesenchymal GBM brain cancer cell-lines used in this work display stem-like characteristics [43] different to NTera2 astrocytes and astrocytes in general. Hence, differences in the patterning dimensions could be expected from this characteristic.

OPI optimal patterning for the FPW1 cell-line produced patterning indices of PAI = 0.67 ± 0.13, SAI = 0.19 ± 0.07, NNI = 0.38 ± 0.03, GQI = 0.48 ± 0.08, and OPI = 0.87 ± 0.10. The RN1 cell-line produced OPI optimal patterning indices of PAI = 0.43 ± 0.04, SAI = 0.16 ± 0.03, NNI = 0.20 ± 0.04, GQI = 0.27 ± 0.04, and OPI = 0.47 ± 0.07. Both FPW1 and RN1 cells conformed to FBS pre-treated parylene-C patterns and were rejected from SiO_2_. However, the FPW1 cell-line demonstrated consistently higher patterning indices. This could be explained by the elliptical morphology of the FPW1 cell-line being better suited to covering parylene-C nodes and strips. Whereas the rounder RN1 cells were less able to extend out long processes and cover the thin parylene-C strips. Patterning of NTera2 astrocytes on parylene-C/SiO_2_ trench networks [37] produced reported patterning indices of PAI = 0.80, SAI = 0.10, NNI = 0.13, GQI = 0.7, and OPI = 0.83; and patterning of NTera2 astrocytes on parylene-C/SiO_2_ networks [40] produced reported patterning indices of PAI = 0.80, SAI = 0.17, NNI = 0.80, GQI = 0.63, and OPI = 1.43. PAI consistently suggests that parylene-C is an attractive substrate for the patterning of glia and GBM cells which are thought to have an astrocyte cell of origin [2,3]. The SAI for NTera2 astrocyte trench network patterning [37] was found to be lower than what was measured in patient-derived mesenchymal GBM cell-lines, this likely reflects that trench networks provide more effective SiO_2_ repulsion.

In this study, optimal patterning was defined using OPI which is a combined measure of single cell patterning as well as cell coverage of the parylene-C pattern. However, if the user wants to maximise parylene-C coverage only, rather than single-cell isolation, it can be observed that choosing a different seeding density or pattern dimension can result in higher values of SAI, PAI or NN1, see Fig 5 - 6 and S1–S4 Figs.

We assessed the functionality of the GBM patterned networks by administering the neurotransmitter ATP. ATP is a common messenger molecule in the nervous system [48] and elicitsCa^2+^ transients in GBM cells [47]. We have previously demonstrated that ATP induces Ca^2+^ transients in NTera2 astrocytes [33,46] and GBM cell-lines [47,49]. The functionality of NTera2 astrocytes patterned on parylene-C/SiO_2_ has been demonstrated by observing the Ca^2+^ transients of non-patterned and patterned NTera2 astrocytes in response to ATP stimulus [33,36]. Jordan *et al* [33] found that NTera2 astrocytes patterned on parylene-C inlayed trenches with D_IN_ = 500 µm produced networks of cells which were unresponsive ATP stimulus. Whereas, NTera2 astrocytes patterned at the D_IN_ = 100 µm by Jordan *et al* [33] were responsive to ATP and produced Ca^2+^ transients with durations ~75 s. Li *et al* [36] also demonstrated the functionality of parylene-C/SiO_2_ patterned NTera2 astrocytes with ATP induced Ca^2+^ responses with durations ~50 s. While there are differences in the durations of the ATP Ca^2+^ transients the overall response of patterned GBM cell-lines was consistent with patterned NTera2 astrocytes.

This work focusses specifically on the patterning of patient-derived mesenchymal GBM cells into regular grid networks, *in vitro*, to facilitate the study of Ca^2+^ communication from the single-cell level to large network scales. However, we acknowledge that the complex tumour microenvironment *in vivo* differs significantly from *in vitro* conditions. There are differences in network dimensions and in the subtle interplay between co-cultures of healthy and glioma cells [50–52]. Therefore, future investigations are required to expand this model further to clarify the discrepancies between GBM cell patterning on parylene-C and the actual growth patterns of GBM *in vivo*.

It should be noted that GBM is a heterogeneous disease with significant intratumoural and intertumoural differences [2]. In this work, we utilised two mesenchymal patient derived cell-lines. Therefore, we would expect there to be differences in the patterning metrics of other patient-derived mesenchymal cell-lines as well as across the other GBM subtypes [43]. We have developed this patterning platform for short term communication studies as the cell-lines we have used pattern within 24 hours. It would be difficult to conduct further long-term studies on viability and functionality beyond this 24 hour window due to the rapid cell division that occurs which disrupts the patterning.

## 5. Conclusion

In this paper we demonstrated how patient-derived mesenchymal glioblastoma brain cancer cells can be reliably patterned into 10 × 10 functional networks on parylene-C/SiO_2_ chips. It was found that a pre-treatment of the parylene-C with PBS or StemPro media did not result in cell conformity to the underlying pattern. In contrast, FBS pre-treatment resulted in preferential attachment of the GBM cells to the underlying parylene-C pattern. GBM cell patterning was assessed over a range of network dimensions, three D_IN_ (50 µm, 76 µm, and 100 µm), three R_N_ (5 µm, 8 µm, and 10 µm), and three cell seeding densities (100 cells mm^-2^, 200 cells mm^-2^, and 300 cells mm^-2^). The OPI optimal patterning conditions for the FPW1 cell-line were a seeding density of 200 cells mm^-2^ with network dimensions of D_IN_ = 100 µm and R_N_ = 10 µm. The patterning indices at these patterning conditions were PAI = 0.67 ± 0.13, SAI = 0.19 ± 0.07, NNI = 0.38 ± 0.03, GQI = 0.48 ± 0.08, and OPI = 0.87 ± 0.10. The RN1 cell-line had OPI optimal patterning conditions at a seeding density 200 cells mm^-2^ with network dimensions of D_IN_ = 76 µm and R_N_ = 10 µm. The patterning indices at these patterning conditions were PAI = 0.43 ± 0.04, SAI = 0.16 ± 0.03, NNI = 0.20 ± 0.04, GQI = 0.27 ± 0.04, and OPI = 0.47 ± 0.07. Unpatterned and patterned FPW1 and RN1 GBM cell-lines responded to ATP stimulation with Ca^2+^ transients of similar durations. The significance of the parylene-C/SiO2 platform presented here, is that it permits the investigation of Ca^2+^ communication within high fidelity, organised, patient-derived GBM mesenchymal grid networks to be more effectively studied. Furthermore, the parylene-C/SiO_2_ patterning platform has the potential to facilitate the GBM scientific community in the testing of candidate therapeutics targeting Ca^2+^ communication.

## Supporting information

S1 Fig Typical patterned FPW1 cell networks on parylene-C/SiO2 substrates seeded at 100 cells mm^-2^. Cell bodies are stained with CMFDA (green) and cell nuclei with NucBlue (blue). All scale bars are 100 µm.
(TIF)

S2 Fig Typical patterned RN1 cell networks on parylene-C/SiO2 substrates seeded at 100 cells mm^-2^. Cell bodies are stained with CMFDA (green) and cell nuclei with NucBlue (blue). All scale bars are 100 µm.
(TIF)

S3 Fig Typical patterned FPW1 cell networks on parylene-C/SiO2 substrates seeded at 300 cells mm^-2^. Cell bodies are stained with CMFDA (green) and cell nuclei with NucBlue (blue). All scale bars are 100 µm.
(TIF)

S4 Fig Typical patterned RN1 cell networks on parylene-C/SiO2 substrates seeded at 300 cells mm^-2^. Cell bodies are stained with CMFDA (green) and cell nuclei with NucBlue (blue). All scale bars are 100 µm.
(TIF)

S1 Table FPW1 Dunn’s test comparison to the OPI optimal dimensions (15k – 10 µm – 100 µm). Asterisks indicate statistically significant differences after Bonferroni correction (p < 0.05).
(PDF)

S2 Table RN1 Dunn’s test comparison to the OPI optimal dimensions (15k – 10 µm – 76 µm). Asterisks indicate statistically significant differences after Bonferroni correction (p < 0.05).
(PDF)

## References

[1] Louis DN, Perry A, Reifenberger G, von Deimling A, Figarella-Branger D, Cavenee WK, et al. The 2016 World Health Organization Classification of Tumors of the Central Nervous System: a summary. Acta Neuropathol. 2016;131(6):803–20. doi: 10.1007/s00401-016-1545-1 27157931

[2] Weller M, Wick W, Aldape K, Brada M, Berger M, Pfister SM. Glioma. Nat Rev Dis Primers. 2015;1:15017. doi: 10.1038/nrdp.2015.17 27188790

[3] Gronseth E, Wang L, Harder DR, Ramchandran R. The role of astrocytes in tumor growth and progression. Astrocyte - Physiology and Pathology. InTech. 2018. doi: 10.5772/intechopen.72720

[4] Rasheed BK, Wiltshire RN, Bigner SH, Bigner DD. Molecular pathogenesis of malignant gliomas. Curr Opin Oncol. 1999;11(3):162–7. doi: 10.1097/00001622-199905000-00004 10328589

[5] Olsen ML, Khakh BS, Skatchkov SN, Zhou M, Lee CJ, Rouach N. New insights on astrocyte ion channels: critical for homeostasis and neuron-glia signaling. J Neurosci. 2015;35(41):13827–35. doi: 10.1523/JNEUROSCI.2603-15.2015 26468182 PMC4604221

[6] Pavlyukov MS, Yu H, Bastola S, Minata M, Shender VO, Lee Y, et al. Apoptotic cell-derived extracellular vesicles promote malignancy of glioblastoma via intercellular transfer of splicing factors. Cancer Cell. 2018;34(1):119-135.e10. doi: 10.1016/j.ccell.2018.05.012 29937354 PMC6048596

[7] Broekman ML, Maas SLN, Abels ER, Mempel TR, Krichevsky AM, Breakefield XO. Multidimensional communication in the microenvirons of glioblastoma. Nat Rev Neurol. 2018;14(8):482–95. doi: 10.1038/s41582-018-0025-8 29985475 PMC6425928

[8] Hong X, Sin WC, Harris AL, Naus CC. Gap junctions modulate glioma invasion by direct transfer of microRNA. Oncotarget. 2015;6(17):15566–77. doi: 10.18632/oncotarget.3904 25978028 PMC4558171

[9] Berridge MJ, Bootman MD, Roderick HL. Calcium signalling: dynamics, homeostasis and remodelling. Nat Rev Mol Cell Biol. 2003;4(7):517–29. doi: 10.1038/nrm1155 12838335

[10] Monteith GR, Prevarskaya N, Roberts-Thomson SJ. The calcium-cancer signalling nexus. Nat Rev Cancer. 2017;17(6):367–80. doi: 10.1038/nrc.2017.18 28386091

[11] Parkash J, Asotra K. Calcium wave signaling in cancer cells. Life Sci. 2010;87(19–22):587–95. doi: 10.1016/j.lfs.2010.09.013 20875431 PMC2974775

[12] Falconnet D, Csucs G, Grandin HM, Textor M. Surface engineering approaches to micropattern surfaces for cell-based assays. Biomaterials. 2006;27(16):3044–63. doi: 10.1016/j.biomaterials.2005.12.024 16458351

[13] Wheeler BC, Brewer GJ. Designing Neural Networks in Culture: Experiments are described for controlled growth, of nerve cells taken from rats, in predesigned geometrical patterns on laboratory culture dishes. Proc IEEE Inst Electr Electron Eng. 2010;98(3):398–406. doi: 10.1109/JPROC.2009.2039029 21625406 PMC3101502

[14] Carter SB. Principles of cell motility: the direction of cell movement and cancer invasion. Nature. 1965;208(5016):1183–7. doi: 10.1038/2081183a0 5331254

[15] Kleinfeld D, Kahler KH, Hockberger PE. Controlled outgrowth of dissociated neurons on patterned substrates. J Neurosci. 1988;8(11):4098–120. doi: 10.1523/JNEUROSCI.08-11-04098.1988 3054009 PMC6569488

[16] Xia Y, Whitesides GM. Soft lithography. Angew Chem Int Ed Engl. 1998;37:550–75. doi: 10.1002/(SICI)1521-3773(19980316)37:5<550::AID-ANIE550>3.0.CO;2-G 29711088

[17] Klebe RJ. Cytoscribing: a method for micropositioning cells and the construction of two- and three-dimensional synthetic tissues. Exp Cell Res. 1988;179(2):362–73. doi: 10.1016/0014-4827(88)90275-3 3191947

[18] Goubko CA, Cao X. Patterning multiple cell types in co-cultures: a review. Materials Science and Engineering: C. 2009;29:1855–68. doi: 10.1016/j.msec.2009.02.016

[19] Hughes MA, Brennan PM, Bunting AS, Cameron K, Murray AF, Shipston MJ. Patterning human neuronal networks on photolithographically engineered silicon dioxide substrates functionalized with glial analogues. J Biomed Mater Res A. 2014;102(5):1350–60. doi: 10.1002/jbm.a.34813 23733444 PMC4243028

[20] Musick K, Khatami D, Wheeler BC. Three-dimensional micro-electrode array for recording dissociated neuronal cultures. Lab Chip. 2009;9(14):2036–42. doi: 10.1039/b820596e 19568672 PMC2818679

[21] Chang TY, Yadav VG, De Leo S, Mohedas A, Rajalingam B, Chen C-L, et al. Cell and protein compatibility of parylene-C surfaces. Langmuir. 2007;23(23):11718–25. doi: 10.1021/la7017049 17915896

[22] Gorham WF. A new, general synthetic method for the preparation of linear poly-p-xylylenes. J Polym Sci A1. 1966;4:3027–39. doi: 10.1002/pol.1966.150041209

[23] Loeb GE, Walker AE, Uematsu S, Konigsmark BW. Histological reaction to various conductive and dielectric films chronically implanted in the subdural space. J Biomed Mater Res. 1977;11(2):195–210. doi: 10.1002/jbm.820110206 323263

[24] Schmidt EM, McIntosh JS, Bak MJ. Long-term implants of Parylene-C coated microelectrodes. Med Biol Eng Comput. 1988;26(1):96–101. doi: 10.1007/BF02441836 3199908

[25] Po-Jui Chen, Rodger DC, Saati S, Humayun MS, Yu-Chong Tai. Microfabricated implantable parylene-based wireless passive intraocular pressure sensors. J Microelectromech Syst. 2008;17(6):1342–51. doi: 10.1109/jmems.2008.2004945

[26] Delivopoulos E, Murray AF, MacLeod NK, Curtis JC. Guided growth of neurons and glia using microfabricated patterns of parylene-C on a SiO2 background. Biomaterials. 2009;30(11):2048–58. doi: 10.1016/j.biomaterials.2008.12.049 19138795

[27] Delivopoulos E, Ouberai MM, Coffey PD, Swann MJ, Shakesheff KM, Welland ME. Serum protein layers on parylene-C and silicon oxide: effect on cell adhesion. Colloids Surf B Biointerfaces. 2015;126:169–77. doi: 10.1016/j.colsurfb.2014.12.020 25555155 PMC4342411

[28] Delivopoulos E, Murray AF, Curtis JC. Effects of parylene-C photooxidation on serum-assisted glial and neuronal patterning. J Biomed Mater Res A. 2010;94(1):47–58. doi: 10.1002/jbm.a.32662 20091707

[29] Unsworth CP, Delivopoulos E, Gillespie T, Murray AF. Isolating single primary rat hippocampal neurons & astrocytes on ultra-thin patterned parylene-C/silicon dioxide substrates. Biomaterials. 2011;32(10):2566–74. doi: 10.1016/j.biomaterials.2010.12.017 21232788

[30] Unsworth CP, Graham ES, Delivopoulos E, Dragunow M, Murray AF. First human hNT neurons patterned on parylene-C/silicon dioxide substrates: combining an accessible cell line and robust patterning technology for the study of the pathological adult human brain. J Neurosci Methods. 2010;194(1):154–7. doi: 10.1016/j.jneumeth.2010.09.022 20933004

[31] Unsworth CP, Holloway H, Delivopoulos E, Murray AF, Simpson MC, Dickinson ME, et al. Patterning and detailed study of human hNT astrocytes on parylene-C/silicon dioxide substrates to the single cell level. Biomaterials. 2011;32(27):6541–50. doi: 10.1016/j.biomaterials.2011.05.041 21641029

[32] Hughes MA, Bunting AS, Cameron K, Murray AF, Shipston MJ. Modulating patterned adhesion and repulsion of HEK 293 cells on microengineered parylene-C/SiO(2) substrates. J Biomed Mater Res A. 2013;101(2):349–57. doi: 10.1002/jbm.a.34329 22847960 PMC4243025

[33] Jordan MD, Raos BJ, Bunting AS, Murray AF, Graham ES, Unsworth CP. Human astrocytic grid networks patterned in parylene-C inlayed SiO2 trenches. Biomaterials. 2016;105:117–26. doi: 10.1016/j.biomaterials.2016.08.006 27521614

[34] Raos BJ, Simpson MC, Doyle CS, Graham ES, Unsworth CP. Evaluation of parylene derivatives for use as biomaterials for human astrocyte cell patterning. PLoS One. 2019;14(6):e0218850. doi: 10.1371/journal.pone.0218850 31237927 PMC6592558

[35] Raos BJ, Doyle CS, Simpson MC, Graham ES, Unsworth CP. Selective PEGylation of parylene-C/SiO2 substrates for improved astrocyte cell patterning. Sci Rep. 2018;8(1):2754. doi: 10.1038/s41598-018-21135-0 29426929 PMC5807449

[36] Li S, Simpson MC, Graham ES, Unsworth CP. Large 10 × 10 single cell grid networks of human hNT astrocytes on raised parylene-C/SiO2 substrates. J Neural Eng. 2019;16(6):066001. doi: 10.1088/1741-2552/ab39cc 31394515

[37] Li S, Graham ES, Unsworth CP. Geometric micro-shapes facilitate trackless connections between human astrocytes. J Neural Eng. 2021;18(3):10.1088/1741-2552/abe7ce. doi: 10.1088/1741-2552/abe7ce 33601342

[38] Murray AF, Delivopoulos E. Adhesion and growth of neuralized mouse embryonic stem cells on parylene-C/SiO2 substrates. Materials (Basel). 2021;14(12):3174. doi: 10.3390/ma14123174 34207642 PMC8226677

[39] Wright D, Rajalingam B, Selvarasah S, Dokmeci MR, Khademhosseini A. Generation of static and dynamic patterned co-cultures using microfabricated parylene-C stencils. Lab Chip. 2007;7(10):1272–9. doi: 10.1039/b706081e 17896010

[40] Wright D, Rajalingam B, Karp JM, Selvarasah S, Ling Y, Yeh J, et al. Reusable, reversibly sealable parylene membranes for cell and protein patterning. J Biomed Mater Res A. 2008;85(2):530–8. doi: 10.1002/jbm.a.31281 17729252 PMC2841044

[41] Tooker A, Meng E, Erickson J, Tai Y-C, Pine J. Biocompatible parylene neurocages. Developing a robust method for live neural network studies. IEEE Eng Med Biol Mag. 2005;24(6):30–3. doi: 10.1109/memb.2005.1549727 16382802

[42] D’Souza RCJ, Offenhäuser C, Straube J, Baumgartner U, Kordowski A, Li Y. Q-cell glioblastoma resource: proteomics analysis reveals unique cell-states are maintained in 3D culture. Cells. 2020;9. doi: 10.3390/cells9020267 PMC7072469 31973233

[43] Stringer BW, Day BW, D’Souza RCJ, Jamieson PR, Ensbey KS, Bruce ZC, et al. A reference collection of patient-derived cell line and xenograft models of proneural, classical and mesenchymal glioblastoma. Sci Rep. 2019;9(1):4902. doi: 10.1038/s41598-019-41277-z 30894629 PMC6427001

[44] Gonzalez RC, Woods RE. Digital Image Processing. 3rd ed. Harlow: Prentice Hall. 2008.

[45] Hochberg Y, Tamhane AC. Multiple Comparison Procedures. Hoboken, NJ: Wiley & Sons. 1987. doi: 10.1002/9780470316672

[46] Haile Y, Fu W, Shi B, Westaway D, Baker G, Jhamandas J, et al. Characterization of the NT2-derived neuronal and astrocytic cell lines as alternative in vitro models for primary human neurons and astrocytes. J Neurosci Res. 2014;92(9):1187–98. doi: 10.1002/jnr.23399 24801011

[47] Strong AD, Indart MC, Hill NR, Daniels RL. GL261 glioma tumor cells respond to ATP with an intracellular calcium rise and glutamate release. Mol Cell Biochem. 2018;446(1–2):53–62. doi: 10.1007/s11010-018-3272-5 29318454 PMC6037622

[48] Burnstock G. Physiology and pathophysiology of purinergic neurotransmission. Physiol Rev. 2007;87(2):659–797. doi: 10.1152/physrev.00043.2006 17429044

[49] Mellor NG, Chung SA, Graham ES, Day BW, Unsworth CP. Eliciting calcium transients with UV nanosecond laser stimulation in adult patient-derived glioblastoma brain cancer cellsin vitro. J Neural Eng. 2023;20(6):10.1088/1741-2552/ad0e7d. doi: 10.1088/1741-2552/ad0e7d 37988746

[50] Sharma P, Aaroe A, Liang J, Puduvalli VK. Tumor microenvironment in glioblastoma: current and emerging concepts. Neurooncol Adv. 2023;5(1):vdad009. doi: 10.1093/noajnl/vdad009 36968288 PMC10034917

[51] Osswald M, Jung E, Sahm F, Solecki G, Venkataramani V, Blaes J, et al. Brain tumour cells interconnect to a functional and resistant network. Nature. 2015;528(7580):93–8. doi: 10.1038/nature16071 26536111

[52] Venkataramani V, Tanev DI, Strahle C, Studier-Fischer A, Fankhauser L, Kessler T, et al. Glutamatergic synaptic input to glioma cells drives brain tumour progression. Nature. 2019;573(7775):532–8. doi: 10.1038/s41586-019-1564-x 31534219

