# Supplementary material for: First cell-patterning of primary, patient-derived mesenchymal glioblastoma brain cancer cells on parylene-C/SiO2 substrates
Source: PLoS One. 2026 Aug 27;21(8):e0352239. doi: 10.1371/journal.pone.0352239 (PMC13521389; doi:10.1371/journal.pone.0352239)
Supplement: Supplementary file 1 [file pone.0352239.s005.pdf]

S1 Table. FPW1 Dunn's test comparison to the OPI optimal dimensions (15k – 10 µm – 100 µm). Asterisks indicate statistically significant differences after Bonferroni correction ($p < 0.05$).

Dimension 1	Dimension 2	z-statistic	p value
15k–10 µm–100 µm	20k–8 µm–76 µm	3.728	0.0676
15k–10 µm–100 µm	20k–8 µm–50 µm	1.488	>0.9999
15k–10 µm–100 µm	20k–8 µm–100 µm	5.326	<0.0001*
15k–10 µm–100 µm	20k–5 µm–76 µm	4.936	0.0003*
15k–10 µm–100 µm	20k–5 µm–50 µm	6.576	<0.0001*
15k–10 µm–100 µm	20k–5 µm–100 µm	3.458	0.1909
15k–10 µm–100 µm	20k–10 µm–76 µm	0.5077	>0.9999
15k–10 µm–100 µm	20k–10 µm–50 µm	2.922	>0.9999
15k–10 µm–100 µm	20k–10 µm–100 µm	1.366	>0.9999
15k–10 µm–100 µm	15k–8 µm–76 µm	3.375	0.2587
15k–10 µm–100 µm	15k–8 µm–50 µm	3.593	0.1146
15k–10 µm–100 µm	15k–8 µm–100 µm	1.56	>0.9999
15k–10 µm–100 µm	15k–5 µm–76 µm	5.286	<0.0001*
15k–10 µm–100 µm	15k–5 µm–50 µm	6.648	<0.0001*
15k–10 µm–100 µm	15k–5 µm–100 µm	3.863	0.0392*
15k–10 µm–100 µm	15k–10 µm–76 µm	1.704	>0.9999
15k–10 µm–100 µm	15k–10 µm–50 µm	1.236	>0.9999
15k–10 µm–100 µm	10k–8 µm–76 µm	2.242	>0.9999
15k–10 µm–100 µm	10k–8 µm–50 µm	6.086	<0.0001*
15k–10 µm–100 µm	10k–8 µm–100 µm	3.301	0.3378
15k–10 µm–100 µm	10k–5 µm–76 µm	6.372	<0.0001*
15k–10 µm–100 µm	10k–5 µm–50 µm	6.359	<0.0001*
15k–10 µm–100 µm	10k–5 µm–100 µm	5.72	<0.0001*
15k–10 µm–100 µm	10k–10 µm–76 µm	1.493	>0.9999
15k–10 µm–100 µm	10k–10 µm–50 µm	2.994	0.9663
15k–10 µm–100 µm	10k–10 µm–100 µm	0.9348	>0.9999
