# Supplementary material for: First cell-patterning of primary, patient-derived mesenchymal glioblastoma brain cancer cells on parylene-C/SiO2 substrates
Source: PLoS One. 2026 Aug 27;21(8):e0352239. doi: 10.1371/journal.pone.0352239 (PMC13521389; doi:10.1371/journal.pone.0352239)
Supplement: Supplementary file 2 [file pone.0352239.s006.pdf]

S2 Table. RN1 Dunn's test comparison to the OPI optimal dimensions (15k – 10 µm – 76 µm). Asterisks indicate statistically significant differences after Bonferroni correction ($p < 0.05$).

Dimension 1	Dimension 2	z-statistic	p value
15k–10 µm–76 µm	20k–8 µm–76 µm	3.654	0.0905
15k–10 µm–76 µm	20k–8 µm–50 µm	4.023	0.0202*
15k–10 µm–76 µm	20k–8 µm–100 µm	3.609	0.1081
15k–10 µm–76 µm	20k–5 µm–76 µm	4.064	0.0169*
15k–10 µm–76 µm	20k–5 µm–50 µm	5.794	<0.0001*
15k–10 µm–76 µm	20k–5 µm–100 µm	3.314	0.3224
15k–10 µm–76 µm	20k–10 µm–76 µm	1.31	>0.9999
15k–10 µm–76 µm	20k–10 µm–50 µm	2.088	>0.9999
15k–10 µm–76 µm	20k–10 µm–100 µm	2.273	>0.9999
15k–10 µm–76 µm	15k–8 µm–76 µm	2.752	>0.9999
15k–10 µm–76 µm	15k–8 µm–50 µm	3.602	0.1108
15k–10 µm–76 µm	15k–8 µm–100 µm	3.508	0.1583
15k–10 µm–76 µm	15k–5 µm–76 µm	6.618	<0.0001*
15k–10 µm–76 µm	15k–5 µm–50 µm	6.236	<0.0001*
15k–10 µm–76 µm	15k–5 µm–100 µm	5.367	<0.0001*
15k–10 µm–76 µm	15k–10 µm–50 µm	0.7583	>0.9999
15k–10 µm–76 µm	15k–10 µm–100 µm	0.2767	>0.9999
15k–10 µm–76 µm	10k–8 µm–76 µm	3.227	0.4388
15k–10 µm–76 µm	10k–8 µm–50 µm	3.434	0.2086
15k–10 µm–76 µm	10k–8 µm–100 µm	3.733	0.0665
15k–10 µm–76 µm	10k–5 µm–76 µm	6.006	<0.0001*
15k–10 µm–76 µm	10k–5 µm–50 µm	6.267	<0.0001*
15k–10 µm–76 µm	10k–5 µm–100 µm	4.857	0.0004*
15k–10 µm–76 µm	10k–10 µm–76 µm	0.2615	>0.9999
15k–10 µm–76 µm	10k–10 µm–50 µm	0.4838	>0.9999
15k–10 µm–76 µm	10k–10 µm–100 µm	1.148	>0.9999
